# Preliminary application of three-dimensional printing in congenital uterine anomalies based on three-dimensional transvaginal ultrasonographic data

**DOI:** 10.1186/s12905-022-01873-0

**Published:** 2022-07-14

**Authors:** Li Wang, Xu-Jiao Chen, Jia-He Liang, Ze-Kai Zhang, Tie-Sheng Cao, Li Zhang

**Affiliations:** 1 Department of Ultrasound Medicine, Tangdu Hospital, the Air Force Medical University, Xi’an, 710038 Shaanxi China; 2 3D Printing Center of the Air Force Medical University, Xi’an, 710038 Shaanxi China

**Keywords:** 3D printing, Transvaginal ultrasonography, Normal uterus, Congenital uterine anomalies

## Abstract

**Background:**

The three-dimensional (3D) printing technology has remarkable potential as an auxiliary tool for representing anatomical structures, facilitating diagnosis and therapy, and enhancing training and teaching in the medical field. As the most available diagnostic tool and it is routinely used as the first approach in diagnosis of the uterine anomalies, 3D transvaginal ultrasonography (3D-TVS) has been proposed as non-invasive “gold standard” approach for these malformations due to high diagnostic accuracy. Despite holding promise of manufacturing 3D printed models based on 3D-TVS data, relevant reports about 3D-TVS derived gynecological 3D printing haven’t been reported to the best of our knowledge. We found an opportunity to explore the feasibility of building 3D printed models for the abnormal uterus based on the data acquired by 3D-TVS.

**Methods:**

The women suspected with congenital uterine anomalies (CUAs) were enrolled in the study. The diagnose of CUAs were made by 3D-TVS scanning and further confirmed under the hysteroscopy examination. One volunteer with normal uterus was enrolled as control. All subjects underwent 3D-TVS scanning for 3D printing data collection. Acquired images were stored and extracted as DICOM files, then processed by professional software to portray and model the boundary of the uterine inner and outer walls separately. After the computer 3D models were constructed, the data were saved and output as STL files for further surface restoration and smoothing. The colors of endometrium and uterine body were specified, respectively, in the print preview mode. Then the uncured photosensitive resin was cleaned and polished to obtain a smooth and transparent solid model after printed models were cooled down.

**Results:**

3D printing models of normal uterus, incomplete septate uterus, complete septate uterus, uterus didelphys and unicornuate uterus were produced on ultrasonographic data of 3D-TVS.

**Conclusions:**

Our research and practice made the first try in modeling CUAs successfully based on ultrasonographic data entirely, verifying that it’s a feasible way to build 3D printed models of high-quality through 3D-TVS scanning.

## Background

In recent years, the technology of three-dimensional (3D) printing has been showed a remarkable potential as an auxiliary tool for representing anatomical structures, facilitating diagnosis and individualized therapy, enhancing training, and teaching in medical area [1–4]. Most of the published studies in the literature within gynecological 3D printing were based on the data derived from magnetic resonance images (MRI) or computerized tomography (CT) [5–7]. However, transvaginal ultrasonography (TVS) as the first-line imaging modality for gynecological disease diagnosing, 3-dimensional TVS (3D-TVS) appears to be the straightforward way for uterine anomaly evaluation and classification [8, 9], holding great promise in 3D modeling, relevant studies about ultrasound derived gynecological 3D printing haven’t been reported.

Congenital uterine anomalies (CUAs) are strongly associated with recurrent pregnancy loss, low birth weight, preterm birth, hypertensive disorders of pregnancy and malpresentation. Prior to conception, women who undergo hysteroscopic metroplasty may have better fertility and pregnancy outcomes [10, 11]. 3D printed models are capable of providing both visual feedback and tangible depth information about anatomic and pathologic severity of CUAs for clinical doctors who can make a precise surgery schedule before operation, and also for the patients who can better understand the pathologic state of uterus.

As the 3D-TVS data with high quality can be acquired for patients with CUAs easily, we made first try to print 3D models completely based on 3D-TVS data in the present study, aiming to find an applicable way and a standard procedure to facilitate ultrasound-based 3D-printing technique in evaluating gynecologic disease.

## Methods

### Study population

This study included four infertile women between the ages of 25 and 33 with regular menstrual cycles, bleeding moderately for 3–5 days with an interval of 28 days. Two of them had previous miscarriages and the other two were diagnosed with ectopic pregnancy and received salpingectomy. All patients underwent hysteroscopy in the Department of Obstetrics and Gynecology at the second Affiliated Hospital of Air Force Military Medical University, which confirmed the diagnosis of incomplete septum uterus, complete septate uterus, uterus didelphys and unicornous uterus, respectively. One female with normal uterus was choose as control. All participants gave their written informed consent after counseling. The study was approved by the institutional review boards of our hospital.

## Ultrasonographic image acquisition

**Fig. 1:**
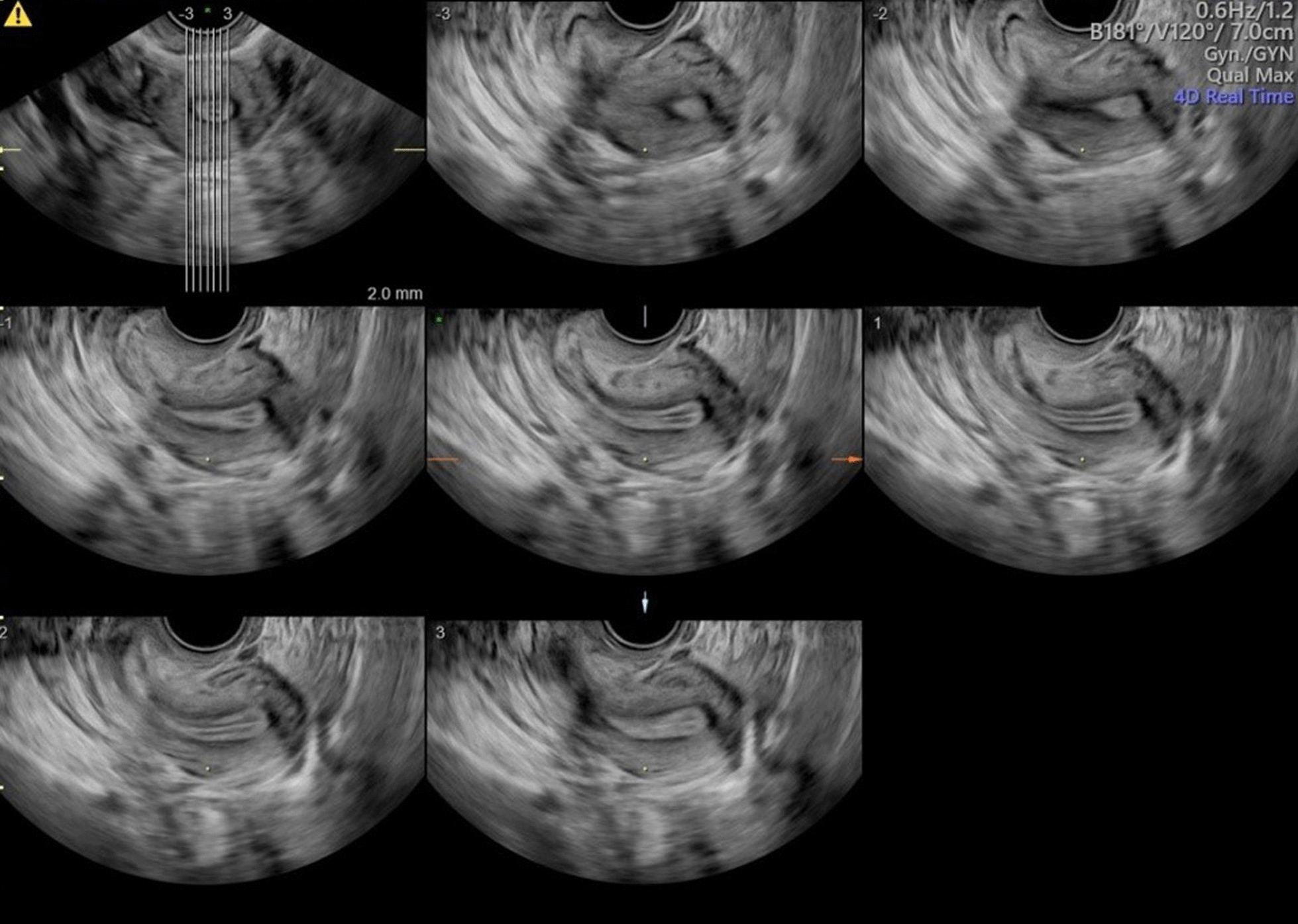
TUI standard mode under TVS scanning

All patients underwent TVS scanning by Voluson E10 (GE Healthcare, Chicago, IL, USA) for 3D ultrasonographic imaging and 3D printing data collection. A RIC 5-9-D ultrasound transducer was adopted to make the diagnosis of CUAs under Render mode firstly and then the TUI (tomographic ultrasound imaging, slices: 7; distance: 2.0 mm) setting was applied in the areas of interests to acquired more precise data for a high-quality 3D model constructing as Fig. 1 showed. The images were stored and extracted as Digital Imaging and Communication in Medicine (DICOM) data form.

## 3D-printing data processing

**Fig. 2:**
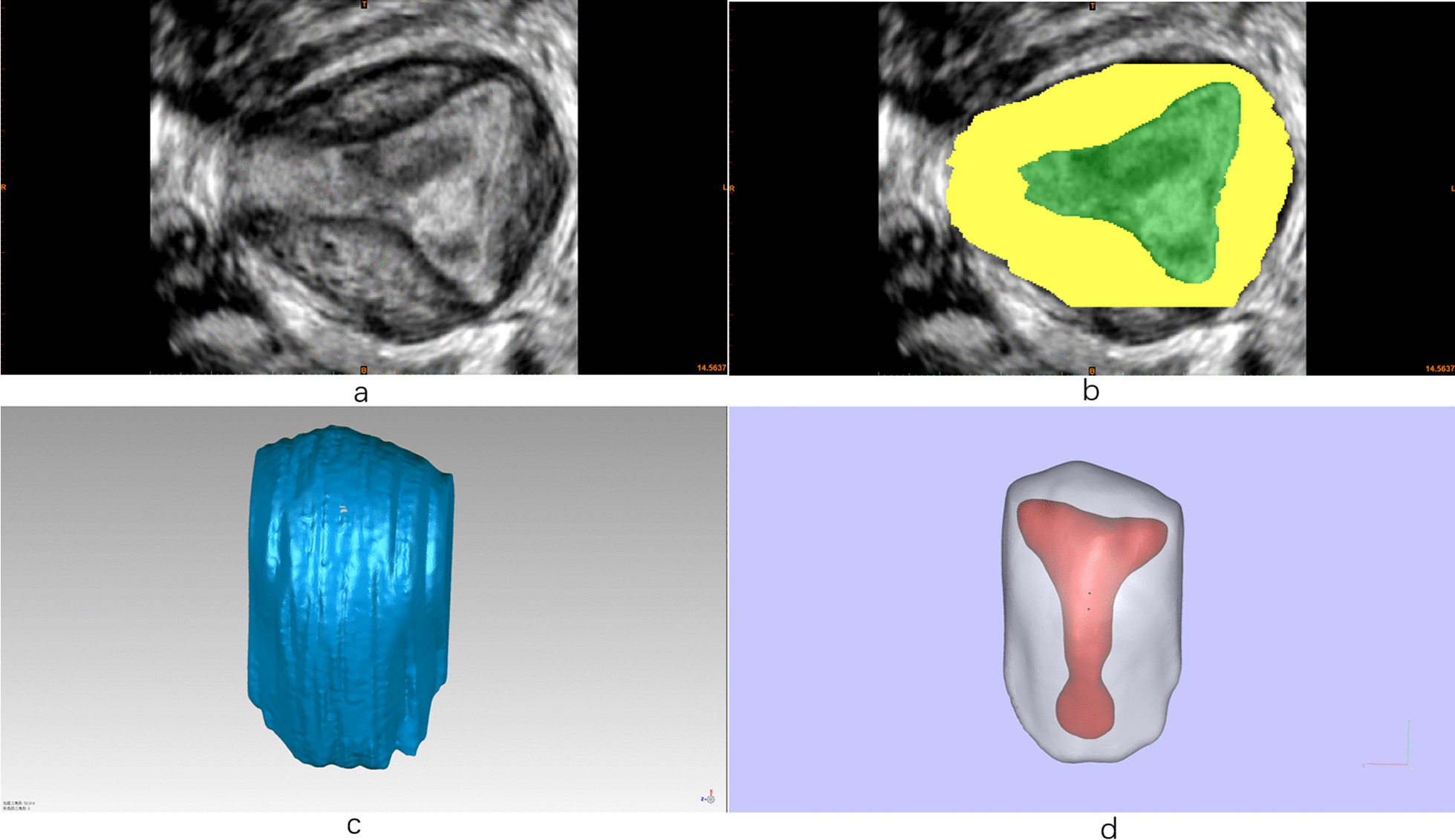
Ultrasonographic data modeling and computer post-processing (**a**) marking the image direction according to the body position, **b** image editing by Mimics, **c** post-processing of image by Geomagics, **d** preview of the colored images by Magics

Firstly, the target DICOM data was opened with Mimics (Research Edition 19.0, Materialise NV, Leuven, Belgium) and the image direction was marked according to the body position. Based on the high resolution ultrasonographic images, the boundary of uterine inner and outer walls can be identified clearly by Mimics, which allowing us to model the uterine inner and outer walls separately. After 3D models were constructed, the data were saved and output as STL file. Geomagics Warp (2017.0.0.111) was applied for surface restoring and smoothing as standard tessellation language (.STL) files of these models were input. Finally, we colored the inner and outer walls differently by Magics 24.0 (V20.0.3.11). The computer processing procedures were showed briefly in Fig. 2.

## Printing models

Import the computer modeling of endometrium and uterine body into the printer system, and the colors can be specified respectively in the mode of print preview. Start printing button. Then the uncured photosensitive resin is cleaned and polished to obtain a smooth and transparent solid model after the temperature of the models was cooled down.

## Results

**Fig. 3:**
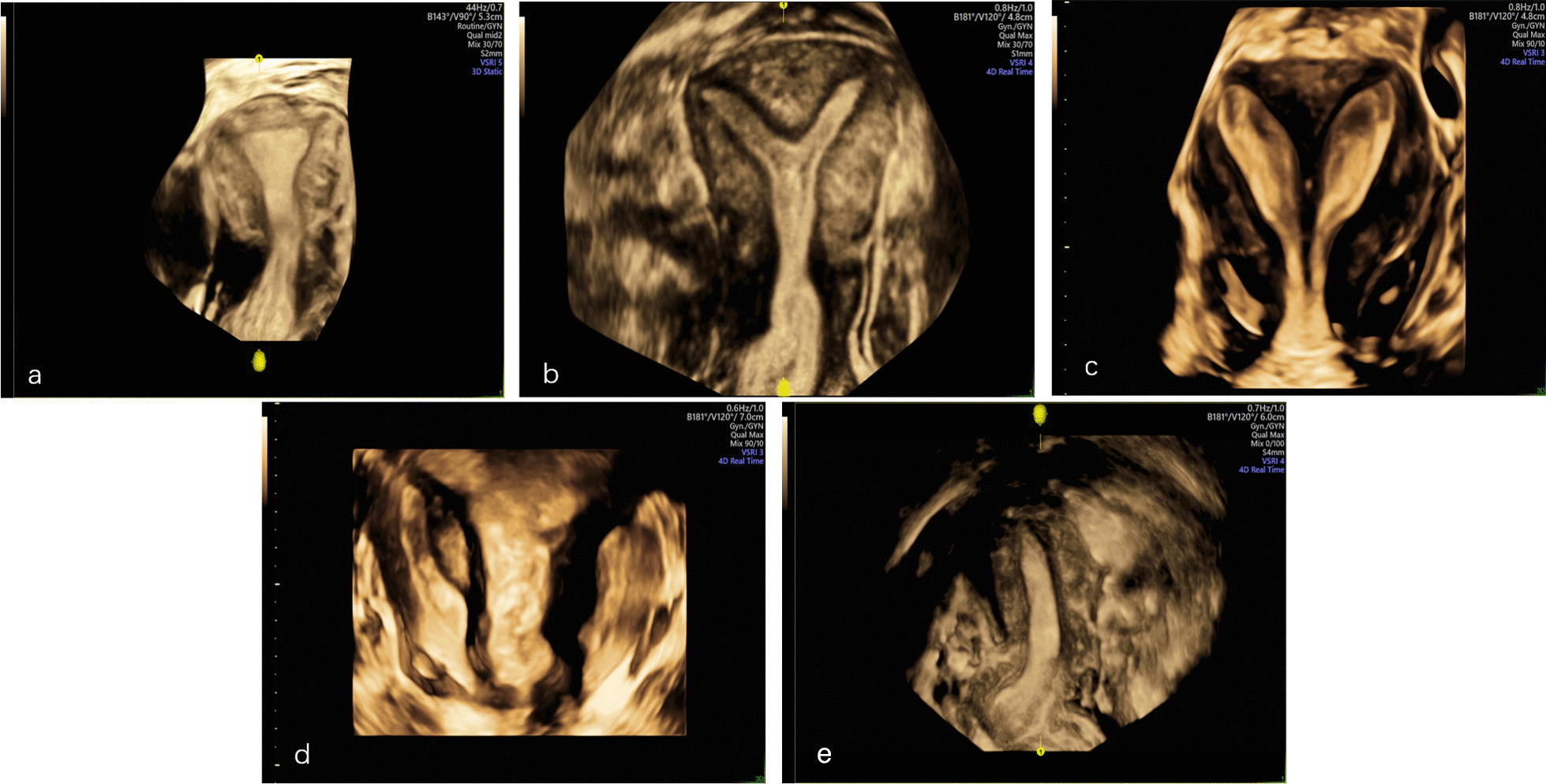
3D-TVS images under Render Mode of normal uterus (**a**), incomplete septum uterus (**b**), complete septate uterus (**c**), uterus didephyl (**d**) and unicornous uterus (**e**)

**Fig. 4:**
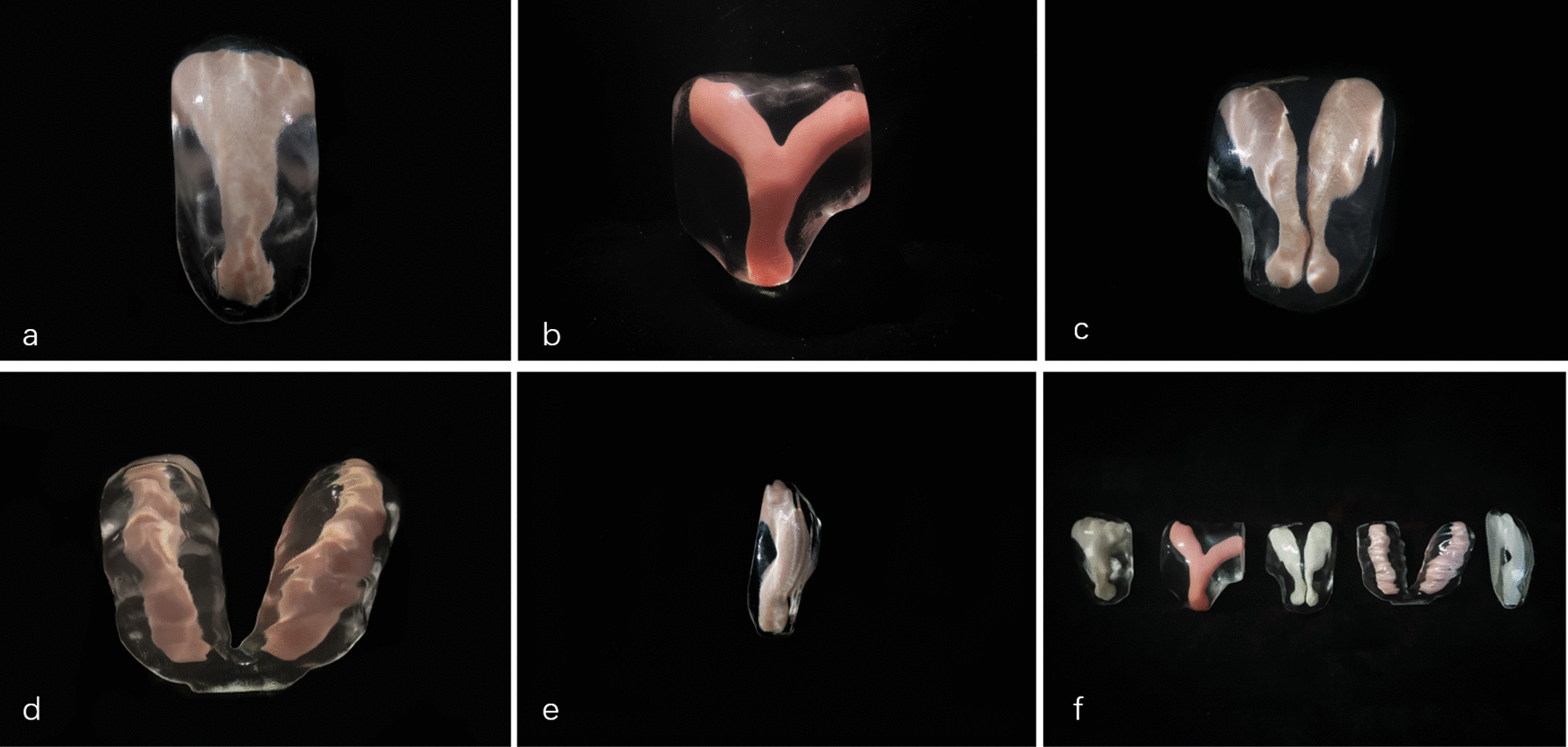
3D printed models of normal uterus (**a**), incomplete septum uterus (**b**), complete septate uterus (**c**), uterus duplex (**d**), unicornous uterus (**e**), and all models’ collection (**f**)

3D-TVS images under Render Mode for normal uterus and CUAs were acquired for all the subjects as Fig. 3 shown. Then the data acquired under TUI mode was input into the computer and the computer modeling process was started subsequently. After labeling the uterine model and model post-processing, the 3D models were printed finally. Uterus was presented as a solid model (1:1 scale) in translucent material with uterine myometrium and uterine septum. Uterine endometrium was printed with colored material in order to get a striking contrast of two parts and highlight the characteristics of the uterine cavity (Fig. 4). It took only several minutes for 3D ultrasonographic data acquisition and data output. Approximated time for data process and printing was 3–4 h.

## Discussion

The first paper in 3D printing within gynecology application was reported by Stitely et al. 2016 about a clinical application of a 3D printed cylindrical tubing connector in 15 s-trimester dilation and evacuation procedures [12]. Meanwhile, Baek et al. provided precise tumor margins information through a 3D printed cervical cancer model, which enabled the surgeon to choose the best alternative for fertility-sparing [13]. Currently, increasing number of 3D printed models applied both in diagnosis and pre-surgical planning of CUAs. Tomlin K et al. made an accurate diagnosis of a female adolescent with unilateral cervical atresia, obstructed hemivagina and ipsilateral renal anomaly with a 3D MRI printed model [8]. Barbosa MZ, et al. built a 3D model to improve reproductive surgery and IVF outcomes including a uterine 3D model for uterine septum correction by hysteroscopy septoplasty [14]. Most papers of gynecological 3D printing were based on MRI or CT acquired data, especially MRI. To the best of our knowledge, there is no publication regarding 3D printing for CUAs based on ultrasound data.

As the reason that CUAs is associated with adverse reproductive outcomes [15], detection of CUAs has been increasing with the advent of 3D TVS. 3D TVS can provide visible evidence of the internal and external contours of the uterus and makes the assessment of uterine morphology with high accuracy when comparing with other commonly used radiological modalities. Then again, 3D ultrasound is a noninvasive, reproducible, and less costly, compared to the MRI, provides more information about the uterus at the coronal plane. Criteria for the classification of uterine anomalies based on 3D ultrasound have been described in the Thessaloniki ESHRE/ESGE consensus on diagnosis of female genital anomalies [16].

The principal aim with 3D printed models was to realize its clinical applications such as performing a better pre-surgical planning for hysteroscopy septoplasty improving surgery accuracy and reducing any iatrogenic risks for the patient as hysteroscopic metroplasty the uterine septum has become the current treatment of choice for patients with septate uterus to keep fertility [17]. 3D models of CUAs were built to enable the surgeon to assess a spatial-location and spatial shape of the septum before surgery, preventing unnecessary incisions. 3D printed models would be good at predicting uterine cavity irregularities for the human reproduction specialist to perform the technique accurately in order to improve pregnancy rate. Moreover, the printed models were available in the operating room for any consult before surgery and for the explanation of the whole procedure to the patient, which considered the model very relevant to her understanding of the disease.

Improvements in computer-aided software, ultrasound imaging resolution and material engineering provided the possibilities to develop 3D printing based on ultrasonographic data. To the best of our knowledge, our research and practice made the first attempt in modeling CUAs based on 3D TVS data, confirming that building 3D printed models with high-quality through 3D-TVS scanning is perfectly workable. The models provided a strong contrast of uterine endometrium with colored material and myometrium with transparent material. It only took us 5 ~ 7 min to complete ultrasound image acquisition and data output, approximated time for data process and printing was 4 ~ 5 h. With the improvement of data post-processing and printing speed, the reduction of model-building time is expected. While time is not necessarily a problem for hysteroscopy septoplasty as an elective surgery.

The main limitation of the present study is the low number of cases. Due to the fact that it was the first try in modeling CUAs based on 3D-TVS data, only four cases with different UCAs were included in the present study, resulting a less convincing, especially a lack of statistical power, of its clinical application value and the superiority of the technology itself. A large sample of subjects need to recruited for further validation.

As we know, building accurate 3D models in medicine requires a learning curve both for the engineers of 3D model reconstruction and the radiologists who acquire 3D images to ensure creating printable file formats [18], which needs multi-field cooperation especially a thorough communication among the clinician, the radiologist and engineer is needed when starting a model reconstruction. Given the extensive applicability of 3D TVS in diagnosing CUAs, our attempt confirmed the feasibility of printing ultrasound-based 3D printed models, may have a positive impact on individualized therapy in the field of gynecology.

## Conclusions

Printing 3D models of CUAs based on ultrasound-derived data is feasible. Our first try provided us more confidence in addressing gynecological diseases. In the future, follow-ups were needed to strength the clinical application of our models especially on reproductive-sparing surgery and pre-assisted reproductive techniques and further applications should be explored.

## Data Availability

All data generated or analyzed during this study are included in this published article.

## Abbreviations

3D: three-dimensional
MRI: magnetic resonance images
CT: computerized tomography
TVS: transvaginal ultrasonography
3D-TVS: 3-dimensional TVS
CUAs: congenital uterine anomalies
TUI: tomographic ultrasound imaging
DICOM: Digital Imaging and Communication in Medicine
STL: standard tessellation language
